# Comparison of Nasopharyngeal Airway Device and Nasal Oxygen Tube in Obese Patients Undergoing Intravenous Anesthesia for Gastroscopy: A Prospective and Randomized Study

**DOI:** 10.1155/2016/2641257

**Published:** 2016-02-22

**Authors:** Qiansong Xiao, Yingying Yang, Yinbin Zhou, Yan Guo, Xing Ao, Ran Han, Jiali Hu, Dongfeng Chen, Chunhui Lan

**Affiliations:** ^1^Department of Gastroenterology, Daping Hospital, Third Military Medical University, Chongqing 400042, China; ^2^Chongqing Medical University, Chongqing 400016, China; ^3^Department of Gastroenterology, Southwest Hospital, Third Military Medical University, Chongqing 400042, China

## Abstract

*Objective*. This prospective and randomized study evaluated the efficacy and safety of the nasopharyngeal airway relative to the nasal oxygen tube in obese patients undergoing painless gastroscopy.* Materials and Methods*. Obese patients (BMI ≥ 28 kg/m^2^; *n* = 260) were randomly and equally apportioned to the nasopharyngeal airway (Group A) or nasal oxygen tube (Group B) group. Three patients were excluded due to failure of insertion of the nasopharyngeal airway. The duration of endoscopy, anesthetic dose, recovery time, and adverse events were recorded. The satisfaction of the anesthetist, physicians, and patient was scored.* Results*. The SpO_2_ reduction was significantly less in Group A than in Group B. Use of a respirator for assisted ventilation occurred significantly less in Group A. The groups were similar regarding mean arterial pressure, heart rate, anesthetic dose, duration of gastroscopy, recovery time, and adverse events. Satisfaction of the physician and anesthetist was greater in Group A; the groups were similar in patient satisfaction.* Conclusions*. Use of the nasopharyngeal airway for obese patients during painless gastroscopy resulted in less SpO_2_ reduction relative to the nasal oxygen tube. Altogether, it is a safe and effective device for obese patients undergoing painless gastroscopy.

## 1. Introduction

Upper gastrointestinal cancers such as gastric and esophageal cancers are among the most lethal malignancies [1], although early detection can greatly reduce morbidity and mortality [2]. Gastroscopy is an effective tool for early detection of upper gastrointestinal cancers. However, routine gastroscopy is associated with several adverse reactions including nausea, vomiting, throat bleeding, and anxiety [2], and patients are reluctant to undergo the procedure. Intravenous anesthesia during gastroscopy increases the comfort of patients during gastroscopy and follow-up examinations [3], thereby improving the early detection rate of precancerous lesions of the upper gastrointestinal tract [4]. Regimens containing the short-acting intravenous anesthetic propofol are most commonly used during gastroscopy, but propofol can inhibit cardiovascular and respiratory function, leading to transient hypotension and bradypnea [5]. General anesthesia without mechanical ventilation is commonly used during gastroscopy and performed with relatively simple monitoring and rescue equipment, and its associated risks are even higher than for general anesthesia administered in the operating room [6].

Obesity is a serious public health problem that affects both adults and children worldwide [7]. During painless gastroscopy performed on obese patients under anesthesia, transient respiratory inhibition can easily occur due to obstruction of the airway by the falling tongue and decreased compliance of the chest wall [8]. Although respiratory depression is transient and may spontaneously recover, it can cause lethal hypoxemia requiring emergent management and thus risks interrupting or even stopping the gastroscopic examination. In addition, because the patient lacks the proper response to hypoxia under anesthesia, it is difficult for the anesthetist to identify the signs of hypoxia promptly. This can lead to serious consequences, such as asphyxia. Therefore, it is important to ensure a smooth flow in the respiratory airway during gastroscopy in patients undergoing intravenous general anesthesia without tracheal intubation [9], particularly for obese patients undergoing painless endoscopy. An effective and safe method to facilitate the flow of the respiratory airway is required.

A nasopharyngeal airway is a simple device that can be conveniently inserted into the supraglottic airway to secure an open passage [7]. It can be connected to a ventilator and thus used in case of emergency in patients with a difficult airway [9]. According to the 2010 American Heart Association Guidelines for Cardiopulmonary Resuscitation and Emergency Cardiovascular Care, a supraglottic airway device (not tracheal intubation) should be the first choice used during emergent resuscitation. The nasopharyngeal airway is an example of a supraglottic airway device used for such conditions [10].

We performed a prospective and randomized study comparing the nasopharyngeal airway and the nasal oxygen tube in obese patients undergoing intravenous anesthesia for painless gastroscopy. Specifically, the 2 devices were evaluated for efficacy, safety, potential adverse events, and satisfaction of the physician, anesthetist, and patient.

## 2. Materials and Methods

The Medical Ethics Committee of Daping Hospital of the Third Military Medical University approved this prospective study. All patients gave their written informed consent. The study was conducted according to the Declaration of Helsinki and registered on 5 February 2015 (registration number: ChiCTR-OPR-15006216, http://www.chictr.org.cn/index.aspx). The study enrolled consecutive obese patients scheduled for painless gastroscopy at the Outpatient Department of Daping Hospital of the Third Military Medical University between March 2015 and July 2015. All subjects conformed to the following inclusion criteria: male or female patients; age between 18 and 80 years; body mass index (BMI) ≥ 28 kg/m^3^; American Society of Anesthesiology (ASA) score I–III physical status; and ability to fill out a survey form and give informed consent. According to the survey of Chinese population, BMI greater than 25 kg/m^2^ was defined as overweight and BMI greater than 30 kg/m^2^ was defined as obesity [11]. Excluded were patients with nasopharyngeal diseases such as polyps, bleeding, trauma, deformity, or inflammation or severe cardiovascular or pulmonary diseases.

The group sample size was predetermined based on the result of a preliminary study of obese patients undergoing gastrointestinal endoscopy. A difference between groups was defined as a difference of 2.54% SpO_2_ with a standard deviation of 5.35%; the failure rate was assumed at 10%. A sample size of 130 subjects per group met the requirement of *α* of 0.05 and power of 0.08 for detecting the difference between groups. The calculations were performed online at http://www.dot05.com/samplesize/1/.

**Figure 1:**
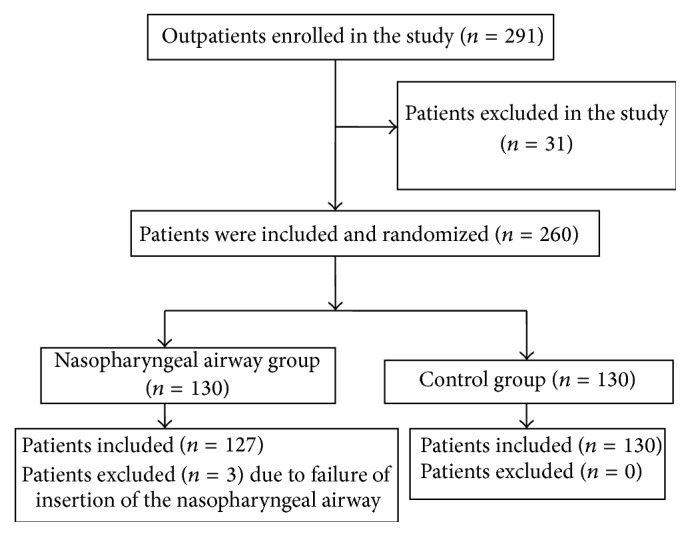
Flow chart showing the inclusion and exclusion criteria for the obese patients in the study.

A total of 291 obese patients were initially enrolled in the study. Thirty-one patients were excluded due to nasopharyngeal diseases or severe cardiovascular and pulmonary diseases. Based on a random number table generated by a computer, the remaining 260 patients were randomly assigned to either the nasopharyngeal airway group using a nasopharyngeal airway during gastroscopy (Group A, *n* = 130) or a control group using a nasal oxygen tube (Group B, *n* = 130). In Group A, 3 patients with stenosis of the nasal cavity were excluded due to failure of insertion of the nasopharyngeal airway (Figure 1).

All patients underwent electrocardiography (ECG) prior to gastroscopy. The patients' ECG, heart rate, height, body weight, and medical history were recorded. All patients were deprived of food for 8 h before the gastroscopy. The patients were given 0.5% oral dimethicone powder (30 mL; Honghe Pharmaceutical, Zigong, China) 30 min before the gastroscopy. After establishing intravenous access, the patients' vital signs were carefully monitored. Oxygen was given through a nasal tube at a rate of 3–5 L/min for 1 min.

Anesthesia was induced by intravenous injections of 1 *μ*g/kg fentanyl (0.02 mL/kg; Yichang Humanwell Pharmaceutical, China) and 2 mg/kg propofol (10 mg/1 mL; Diprivan, AstraZeneca, United Kingdom). Additional 0.5 mg/kg propofol was given as needed during gastroscopy. Intravenous anesthesia was administered by an anesthesiologist blinded to the study. Patients undergoing sedated endoscopy were evaluated according to the Ramsay Sedation Scale (RSS) [12] as follows: 1, patient was anxious and agitated or restless or both; 2, patient was cooperative, orientated, and tranquil; 3, patient responded to commands only; 4, patient had a brisk response; 5, patient had a sluggish response; and 6, patient had no response. For all patients, the RSS score was maintained at >4 during gastroscopy. Gastroscopy was performed by 2 experienced physicians after induction of anesthesia, using an electronic gastroscope (GIF-H260, Olympus, Japan). After gastroscopy, all drugs were immediately stopped, and the nasopharyngeal airway was removed after the patient recovered from anesthesia.

For patients undergoing nasopharyngeal airway (Group A), a disposable nasopharyngeal airway of appropriate size (I.D. 6.0 mm, 6.5 mm, 7.0 mm, 7.5 mm, or 8.0 mm) was selected by examining the outer diameter of nasopharyngeal airway lumen and patients nostrils. The airway that is large enough but can be easily inserted through the nostril was selected. The surface of the airway was lubricated with lidocaine gel (Kangye Pharmaceutical, Handan, China) before insertion. The curved end of the airway was inverted towards the hard palate or the roof of the mouth and placed at the posterior nasopharynx. After the distal end of the device was secured, an oxygen tube was inserted into the airway.

Each patient's mean arterial pressure (MAP), heart rate, and SpO_2_ were measured and recorded at 2 min before endoscopic procedure (before gastroscopy), when gastroscopy was inserted into the gastric antrum (during gastroscopy), and at 5 min after the patient regained consciousness (after gastroscopy). The duration of endoscopy, the doses of anesthetics, recovery time from anesthesia, and adverse events such as body movement, regurgitation and aspiration, bronchospasm, and cough were recorded. The satisfaction of the anesthetist, physician, and patient was assessed using a 10-point scale as follows: poor, 1–4; fair, 5–7; good, 8–10.

### 2.1. Statistical Analysis

Statistical analyses were performed using SPSS 13.0 (SPSS, Chicago, IL, USA). Numerical data are presented as mean ± standard deviation and compared using Student's *t*-tests. Qualitative data are expressed as *n* (%) and compared using the chi-squared test. A *P* value < 0.05 was considered statistically significant.

## 3. Results and Discussion

### 3.1. Baseline Characteristics

Between Group A (nasopharyngeal airway) and Group B (nasal oxygen tube) there were no significant differences in age, gender, or BMI (Table 1). There was also no statistical difference in the percentage of underlying medical conditions such as ECG abnormality, hypertension, history of surgery, diabetes, or coronary diseases. There was no significant difference in the purpose of upper GI endoscopy between the two groups.

### 3.2. Hemodynamic Parameters

**Figure 2:**
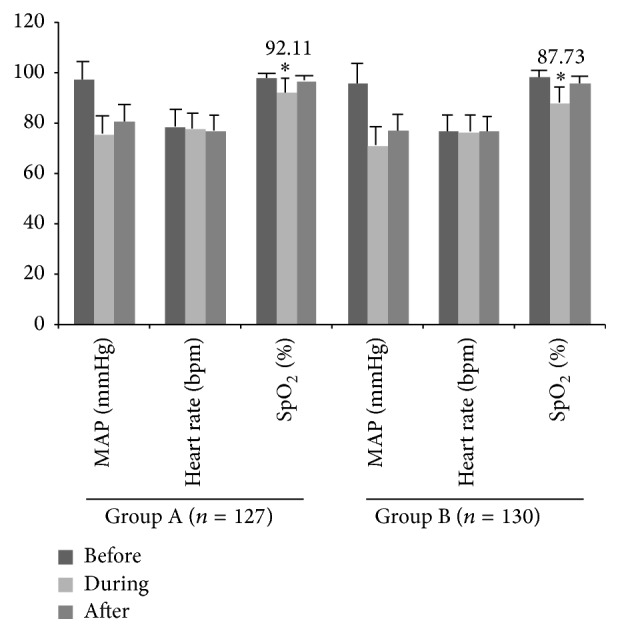
MAP, heart rate, and SpO_2_ of Group A and Group B before, during, and after gastroscopy. ^*∗*^Significant difference between the 2 groups.

Before gastroscopy, between the 2 groups there were no significant differences in MAP, heart rate, or SpO_2_ (Figure 2). In both groups, the MAP and heart rate transiently decreased after anesthesia induction. Also between the groups, there were no significant differences in MAP or heart rate during or after the gastroscopy (*P* > 0.05).

For both groups, the SpO_2_ levels were significantly lower during gastroscopy compared with the baseline values (*P* < 0.05). The SpO_2_ decreased from 98.14% at the baseline to 92.11% during gastroscopy and to 96.70% after gastroscopy in Group A and decreased from 98.19% at the baseline to 87.73% during gastroscopy and to 96.10% after gastroscopy in Group B. The amount of reduction in SpO_2_ during and after gastroscopy from baseline in Group A (6.03% and 1.44%, resp.) was significantly less than in Group B (10.46% and 2.09%, resp.) (*P* = 0.009 and *P* = 0.046, resp.). Patients in Group A required emergent management (raising the mandible, hyperbaric oxygen supply, and use of the respirator) less frequently than did those in Group B (*P* < 0.05, Table 2).

### 3.3. Doses of Anesthetics, Duration of Gastroscopy, and Recovery Time

Between the 2 groups, there were no significant differences in doses of anesthetics, duration of gastroscopy, or recovery time (Table 3).

### 3.4. Satisfaction of Physician, Anesthetist, and Patient

The satisfaction of the physician and anesthetist was significantly greater for procedures using the nasopharyngeal airway (Group A) than for those using the nasal oxygen tube (Group B, Table 4). The satisfaction of the patients in these groups was statistically similar.

### 3.5. Adverse Events

The incidence of adverse events such as body movement, regurgitation and aspiration, bronchospasm, and cough was low and statistically similar in both groups (Table 5).

The nasopharyngeal airway can prevent airway obstruction caused by the falling tongue and reduce air flow resistance, thus facilitating spontaneous breathing by the patient [13]. In addition, the nasopharyngeal airway can reduce injury to the mucosa of the nasal cavity caused by repeated suction of sputum, especially for patients with viscous sputum. It has been reported that use of the nasopharyngeal airway during general anesthesia can effectively and safely prevent airway obstruction [14]. However, it remains unclear whether the nasopharyngeal airway is beneficial during gastroscopy for patients given general anesthesia.

In this prospective study, we compared the efficacy and safety of the nasopharyngeal airway with those of the nasal oxygen tube in obese patients to whom intravenous anesthesia for gastroscopy was administered. We found that the reduction in SpO_2_ during and after gastroscopy compared with the baseline was significantly less in patients in whom the nasopharyngeal airway was used, and emergent management was also required less frequently. There were no significant differences in the rates of adverse events between the 2 groups. Our study suggests that the nasopharyngeal airway is an effective and safe device for obese patients undergoing intravenous anesthesia for gastroscopy.

The fact that SpO_2_ reduction during and after gastroscopy was significantly less in the group treated with the nasopharyngeal airway indicates that this device is more able than the nasal oxygen tube to reduce airway obstruction and facilitate patient's breathing. In addition, patients for whom the nasopharyngeal airway was used required significantly less emergent management. Simple management such as raising the mandible and supplying hyperbaric oxygen can reduce hypoxia. For patients in the nasopharyngeal airway group, the simple respirator was used significantly less often and there was no requirement for the ventilator, thus reducing the rate of emergent management that interrupts or stops the gastroscopic examination. The lesser need for emergent management or interrupted gastroscopic examination may be why the satisfaction ratings given by the physician and anesthetist for this group were higher.

Even though the use of the nasopharyngeal airway requires extra operative time compared with the nasal oxygen tube, the duration of gastroscopy was not significantly different between the 2 groups, since insertion of the nasopharyngeal airway is simple and easy. Thus, insertion of the nasopharyngeal airway did not result in an increase in the anesthetic dose or recovery time. In addition, there were no significant differences between the 2 groups with regard to MAP or heart rate during and after the gastroscopy, suggesting that the nasopharyngeal airway did not cause a significant change in blood pressure or heart rate.

Since the soft nasal tissue is easily damaged, violent insertion of the nasopharyngeal airway can result in injury to the upper nasal cavity [15]. In the present study, failure of insertion of the nasopharyngeal airway occurred in 3 patients due to stenosis of the nasal cavity. Although the failure rate was low (2.3%, 3/130), care should be taken to avoid damage to the nasal cavity in patients with stenosis of the nasal cavity. In the present study, no nasal bleeding caused by nasopharyngeal airway device occurred, since the anesthetist stopped the insertion of the device when the discomfort of the patient occurred.

In the present study, we found that the incidence of adverse events for patients in the nasopharyngeal airway group was very low. Adverse events occurred in only 7 patients (5.51%), consisting of body movement, regurgitation and aspiration, and cough, 2, 1, and 4 cases, respectively. The occurrence of adverse events in patients in the nasopharyngeal airway group was comparable to that of patients in the nasal oxygen tube group. This suggests that use of the nasopharyngeal airway was well tolerated by patients, perhaps because the nasopharyngeal airway is easy to operate, without the assistance of any special instruments, and its soft surface is lubricated with lidocaine gel. Therefore, if gentle insertion of the nasopharyngeal airway is ensured, its use is feasible for painless gastroscopy.

## 4. Conclusions

In summary, this prospective study evaluated the efficacy and safety of the nasopharyngeal airway relative to those of the nasal oxygen tube in obese patients undergoing intravenous anesthesia for gastroscopy. We found that patients in the nasopharyngeal airway group experienced less reduction in SpO_2_ and required less emergent treatment during gastroscopy. These results indicate that use of the nasopharyngeal airway can facilitate air flow of the respiratory airway. In addition, the rates of hemodynamic fluctuation and adverse events were comparable for the 2 groups, supporting our conclusion that the nasopharyngeal airway is a safe device for obese patients undergoing intravenous anesthesia for gastroscopy.

## Figures and Tables

**Table 1:** Baseline characteristics of patients in Groups A and B.

	Group A	Group B	*P* value
Subjects, *n*	127	130	
Age, y	44.02 ± 11.68	45.92 ± 11.07	0.503
Gender, M/F	88/39	91/39	0.505
BMI	30.32 ± 2.14	30.21 ± 2.26	0.903

Underlying medical conditions, *n* (%)			
ECG abnormality	2 (1.57%)	1 (0.77%)	0.491
Hypertension	7 (5.51%)	6 (4.62%)	0.482
History of surgery	3 (2.36%)	1 (0.77%)	0.302
Diabetes	3 (2.36%)	2 (1.54%)	0.489
Coronary disease	1 (0.79%)	1 (0.77%)	0.745

The purpose of upper GI endoscopy, *n* (%)			
Abdominal pain	39 (30.70%)	43 (33.08%)	0.684
Nausea and vomiting	12 (9.45%)	9 (6.92%)	0.460
Bloating	15 (11.81%)	13 (10.00%)	0.641
Haemorrhage	9 (7.09%)	12 (9.23%)	0.530
Loss of appetite	26 (20.47%)	22 (16.92%)	0.465
Diarrhea	8 (6.30%)	10 (7.69%)	0.662
Examination	18 (14.17%)	21 (16.15%)	0.658

**Table 2:** Emergent management in the treatment groups^*∗*^.

	Group A	Group B	*χ* ^2^	*P* value
Subjects, *n*	127	130	—	—
Emergent management	17 (13.39%)	41 (31.54%)	80.411	<0.001
Raising the mandible	14 (11.02%)	41 (31.54%)	88.251	<0.001

Hyperbaric oxygen supply	17 (13.39%)	40 (30.77%)	82.447	<0.001
Use of simple respirator	10 (7.90%)	25 (19.2%)	138.068	<0.001
Use of ventilator	0 (0.00%)	1 (0.01%)	—	—

^*∗*^ *n* (%), unless indicated otherwise.

**Table 3:** Anesthetic dose, duration of gastroscopy, and recovery time of the treatment groups.

	Group A	Group B	*t*	*P* value
Propofol, mL	12.76 ± 3.50	12.50 ± 3.49	0.570	0.972
Duration of gastroscopy, s	304.53 ± 70.97	303.46 ± 72.49	0.120	0.359
Recovery time, s	140.16 ± 59.84	139.74 ± 69.11	0.052	0.065

**Table 4:** Satisfaction of physician, anesthetist, and patient with the 2 treatments.

	Group A			Group B			*P* value
	Good	Fair	Poor	Good	Fair	Poor	
Physician	109 (85.83%)	18 (14.17%)	0 (0%)	97 (74.62%)	33 (25.38%)	0 (0%)	0.018
Anesthetist	115 (90.55%)	12 (9.45%)	0 (0%)	99 (76.15%)	31 (23.84%)	0 (0%)	0.002
Patient	112 (88.19%)	15 (11.81%)	0 (0%)	121 (93.07%)	9 (6.92%)	0 (0%)	0.129

**Table 5:** Adverse events in Group A and Group B, *n* (%).

	Group A	Group B	*χ* ^2^	*P* value
Total events	7 (5.51%)	12 (9.23%)	1.298	0.184
Body movement	2 (1.57%)	4 (3.08%)	0.636	0.684
Regurgitation and aspiration	1 (0.79%)	2 (1.54%)	0.575	0.509
Bronchospasm	0 (0.00%)	1 (0.77%)	—	—
Cough	4 (3.15%)	5 (3.85%)	0.761	0.515
